# On the design of power gear trains: Insight regarding number of stages and their respective ratios

**DOI:** 10.1371/journal.pone.0198048

**Published:** 2018-06-01

**Authors:** Harrison L. Bartlett, Brian E. Lawson, Michael Goldfarb

**Affiliations:** Department of Mechanical Engineering, Vanderbilt University, Nashville, TN, United States of America; Chongqing University, CHINA

## Abstract

This paper presents a formulation for selecting the stage ratios and number of stages in a multistage transmission with a given desired total transmission ratio in a manner that maximizes efficiency, maximizes acceleration, or minimizes the mass of the transmission. The formulation is used to highlight several implications for gear train design, including the fact that minimizing rotational inertia and mass are competing objectives with respect to optimal selection of stage ratios, and that both rotational inertia and mass can often be minimized by increasing the total number of stages beyond a minimum realizable number. Additionally, a multistage transmission will generally provide maximum acceleration when the stage ratios increase monotonically from the motor to the load. The transmission will have minimum mass when the stage ratios decrease monotonically. The transmission will also provide maximum efficiency when the corresponding stages employ constant stage ratios. This paper aims to use this optimization formulation to elucidate tradeoffs between various common objectives in gear train design (efficiency, acceleration, and mass).

## Introduction

Electric motors are commonly employed to actuate drive systems, but generally provide power in a high speed, low torque power regime relative to the drive applications they are intended to actuate. In order to address this disparity, gear transmissions are commonly employed to transform the high speed, low torque output power of the motor to the requisite higher torque, lower speed power regime of the drive system. Such gear transmissions are commonly of the multistage [1, 2] or planetary types [3, 4]. This paper describes methods of optimally selecting gear trains of the multistage type. Drive systems with large transmission ratios (e.g., on the order of 100:1) are common in modern electric motor-driven machines. Such transmissions are typically split into multiple stages for the purposes of efficient packaging and practical constraints on transmission size. When considering the design of a multistage transmission of a given overall transmission ratio, a designer must determine the most appropriate number of stages, and the stage ratio associated with each. This paper considers the problem of selecting number of stages, and stage ratios, from the perspective of either maximizing transmission efficiency, maximizing rotational acceleration capabilities, or minimizing mass.

The general objective of optimal gear transmission design is not new–various approaches have been presented by others previously, such as those described in [1, 2, 5–25]. Of the works most relevant to this paper, among the earliest is [1], in which the authors minimized the rotational inertia reflected onto the motor shaft of a two-stage transmission with a pre-specified total transmission ratio, using the assumption of size-invariant pinions for each stage. The optimal stage ratios for multistage transmissions with more than two stages was also presented in that paper, although in a graphical manner. An iterative, numerical method for optimizing multistage transmissions with respect to minimizing transmission rotational inertia was presented in [2, 6, 8]. Numerical multi-criterion optimization methods for transmissions were also presented in [9–16], which consider objective functions such as gear train volume and efficiency. Various optimization algorithms have also been described for the problem of choosing stage ratios of a gear train in which each gear is constrained to have a predetermined number of teeth [17, 18]. In terms of selecting the number of stages in a gear train, [2] provides an analytical solution for the number of stages that should be chosen to minimize the reflected inertia of a transmission.

This paper presents a formulation for the design of a multistage gear train that selects stage ratios in a manner that maximizes transmission efficiency, maximizes rotational acceleration, or minimizes mass for a given desired total transmission ratio. The problem is formulated as a constrained multivariate optimization problem for any number of stages *n*, and can be used with a wide variety of stage scaling criteria. The scaling criterion employed in this manuscript is intended for power gear trains, and, as a result, is derived to provide constant tooth stress across the stages. In addition to presenting optimal stage ratio formulations, the paper describes some implications of these formulations with respect to gear train design, specifically with respect to how these performance characteristics relate to number of stages and variation in stage ratios.

Unlike prior related works [1–23], this paper provides formulations for the optimal efficiency, acceleration, and mass, respectively, as a function of number of stages and stage ratios, for a given total transmission ratio. The authors note that, this paper also provides a topological context for the solution presented in [2]. Specifically, [2] does not provide information regarding the local curvature around the optimal solution, and thus does not provide information regarding the sensitivity of the optimal solution as a function of the number of stages. Without this information, a designer is unable to assess the trade-offs entailed in a sub-optimal solution, which is of fundamental importance in the design process. In fact, the relevant objective function, as constructed herein, is quite flat around the optimal solution for most applications, and as such, employing the solution presented in [2], although mathematically correct, would often result in an infeasibly large number of stages. By examining the topology of the objective function, the (maximum rotational acceleration) solution presented here enables a more knowledgeable selection of stages, which can provide near-optimal performance in a feasible transmission configuration. This paper additionally examines the optimal design of gear trains with respect to multiple different objective functions, and subsequently illustrates the inherent tradeoff between mass and inertia in the gear train design process. This work allows a designer to select gear train parameters with knowledge of how design decisions might affect multiple design objectives.

## Formulation

Let *n* be the number of stages in a transmission with a total transmission ratio (i.e., reduction ratio) of *N*:1, and let the respective stage ratio, *s*_*i*_, be the transmission ratio corresponding to the *i*^th^ stage of the transmission (where the stage index *i* increases from the motor to the load). The *n* stage ratios must multiply to equal the total desired transmission ratio *N*, as expressed by the following constraint equation:

$$\left(\prod_{i=1}^{n}s_{i}\right)-N=0$$ (1)

**Fig 1:**
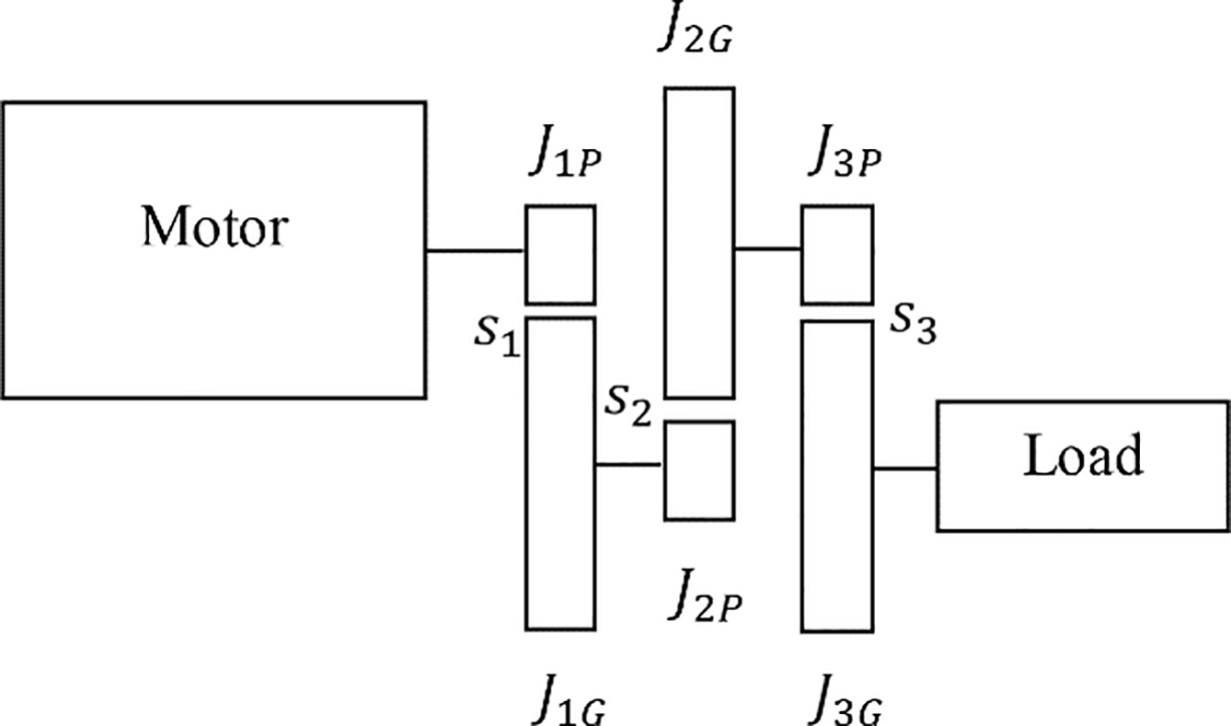
Schematic of a multistage transmission consisting of a motor, transmission, and load. The inertia of a gear or pinion is denoted as *J* with the first subscript indicating the stage of the transmission. The second subscript denotes if a component is a gear (*G*) or pinion (*P*). The stage ratio of the *i*^th^ stage is denoted by *s*_*i*_.

In the following sections, expressions for the transmission efficiency, maximum achievable acceleration, and transmission mass are developed as functions of the stage ratios. A representative 3-stage (*n* = 3) transmission can be seen in Fig 1 which defines key design variables.

### Maximum gear train efficiency

Gear train efficiency is important in applications in which power consumption or maximum output torque are primary concerns. The total transmission efficiency, *η*_*T*_, is calculated as the product of the individual stage efficiencies, *η*_*i*_ (2).

$$\eta_{T}=\prod_{i=1}^{n}\eta_{i}$$ (2)

The efficiency of a single stage of a spur or helical gear train can be calculated as described in [26]:

$$\eta_{i}=1-\mu K-\mu Ks_{i}$$ (3)

where *μ* is the coefficient of friction between the gears (typically between 0.05 and 0.15) and *K* is a constant containing information about the gear geometry as follows:

$$K=\frac{\pi }{Z_{1}}\frac{1}{\cos(\beta )}\varepsilon_{\alpha }\Lambda$$ (4)

where *Z*_1_ is the number of teeth on the pinion in the mesh, *β* is the base helix angle (for helical gears), *ε*_*α*_ is the profile contact ratio, and Λ is the loss factor as described in [26]. These efficiency equations of a single stage (3–4) are a reorganization of equation (26) in [26]. The set of *s*_*i*_ that maximizes (2), subject to the constraint (1), will provide the maximum possible efficiency for the multistage transmission.

### Maximum achievable acceleration

The maximum (no-load) acceleration per unit torque of the drive system (normalized to the first pinion of the gear train) can be described by:

$$\frac{\ddot{\theta }}{\tau_{m}}=\frac{\eta_{T}J_{1P}}{J_{m}}$$ (5)

where *τ*_*m*_ is the motor torque, $\ddot{\theta }$ is the motor acceleration, *J*_*m*_ is the transmission inertia reflected onto the motor, and *J*_1*P*_ is the inertia of the first stage pinion. The size of the first pinion in the gear train is fully determined by the torque and speed of the motor, and it is therefore convenient to normalize the reflected inertia by the inertia of the first stage pinion, (*J*_1*P*_). This inertia normalization ($\frac{J_{m}}{J_{1P}}$) essentially removes absolute sizing and choice of gear material from the problem.

In order to maximize the acceleration capabilities of a transmission, the right hand side of (5) must be maximized with respect to the stage ratios. However, the reflected inertia of the transmission onto the motor must also be expressed in terms of the stage ratios of the transmission. This problem has been treated in other works [1, 2, 5, 6, 25] and can be written as follows for an *n*-stage transmission where *n*>1:

$$J_{m}=J_{1P}+\sum_{i=1}^{n-1}\left[\left(J_{iG}+J_{\left(i+1\right)P}\right){\left(\frac{1}{\prod_{j=1}^{i}s_{j}}\right)}^{2}\right]+J_{nG}{\left(\frac{1}{\prod_{k=1}^{n}s_{k}}\right)}^{2}\begin{array}{cc} for & n>1 \end{array}$$ (6)

where *J*_*iP*_ is the rotational inertia of the *i*^th^ pinion, and *J*_*iG*_ is the rotational inertia of the *i*^th^ gear. The set of *s*_*i*_ that maximizes (5), subject to the constraint (1), results in a multistage gear train that will provide the maximum achievable acceleration at the output.

It should be noted that several prior works consider the problem of minimizing rotational inertia [1, 2, 5, 6, 25] and the corresponding implications for maximizing output acceleration. The formulation presented here considers the objective of maximizing rotational acceleration by considering both inertia and efficiency, since both are functions of the stage configuration, and both affect output acceleration.

### Minimum gear train mass

The normalized mass of an *n*-stage transmission, *M*, can be calculated by adding the mass of the pinions and gears of each stage (*m*_*iP*_ an*d m*_*iG*_, respectively) and normalizing this quantity by the mass of the first stage pinion:

$$M=\frac{\sum_{i=1}^{n}\left(m_{iP}+m_{iG}\right)}{m_{1P}}$$ (7)

This normalization is performed to remove absolute sizing and material selection from the problem. The set of *s*_*i*_ that minimizes (7), subject to the constraint (1), results in a multistage gear train with the minimum possible transmission mass.

### Scaling for constant gear stress

Although normalization of the respective objective functions by the first-stage pinion removes overall scale and material selection from the optimization problem, a relationship is still required to inform the manner in which each successive pinion (i.e., gearset) should be scaled relative to the first. A reasonable assumption in this regard is that all gears are formulated from the same material, and each is stressed to the same maximum bending stress (i.e., each gear is designed to operate with the same factor of safety). A scaling relation can be derived by considering a standard model of bending stress within a gear tooth [27]:

$$\sigma =\frac{2\tau P}{dFY}$$ (8)

where *τ* is the pinion torque, *P* is the diametral pitch, *F* is the face width, *d* is the pitch diameter, and *Y* is the Lewis form factor. For simplification, it is assumed that pinions of successive stages have the same Lewis form factor (*Y* remains constant across successive stages). This assumption is reasonable given that pinions typically employ the minimum number of possible teeth, and gears with similar numbers of teeth have similar Lewis form factors. Imposing constant stress at the pinions of two successive stages of a gear train leads to the following expression:

$$\frac{2\tau_{i}P_{i}}{d_{i}F_{i}Y}=\frac{2s_{i}\tau_{i}P_{i+1}}{d_{i+1}F_{i+1}Y}$$ (9)

where the “*i*” subscript indicates the first of the two stages, a “*i*+1” subscript indicates the second of the two stages, and where *s*_*i*_ is the ratio of the first of these two stages. Assuming that pinions of successive stages have the same number of teeth and noting that the diametral pitch is the number of gear teeth divided by the pitch diameter, the following scaling ratio is determined:

$$\frac{d_{i+1}}{d_{i}}=\frac{P_{i}}{P_{i+1}}$$ (10)

A simple approximation of the relationship between diametral pitch (*P*) and face width of a gear (*F*) can also be assumed such that the face width of a gear is inversely proportional to the gear’s diametral pitch ($F\propto \frac{1}{P}$), which provides a reasonable approximation of accessible geometric data for commercially available gears. This inverse proportionality assumption leads to a secondary gear scaling ratio between face width and pitch:

$$\frac{F_{i+1}}{F_{i}}=\frac{P_{i}}{P_{i+1}}$$ (11)

Substituting (10) and (11) in to (9) and isolating the stage ratio, *s*_*i*_, yields the following pinion scaling relationship for two successive stages in a spur gear train such that each pinion is subject to the same stress:

$$\frac{F_{i+1}}{F_{i}}=\frac{d_{i+1}}{d_{i}}=\frac{P_{i}}{P_{i+1}}=s_{i}{}^{\frac{1}{3}}$$ (12)

The expression presented in (12) matches the empirical scaling recommendations described in [2]. Note that this expression assumes the meshing gears and pinions have the same face widths. It should also be noted that this assumption neglects the effect of speed (i.e., pitch-line velocity) on gear stress, but in the context of the optimization, neglecting such effects are not expected to substantially affect the solution.

The relationships in (12) must be expressed such that the ratio of the mass and inertia of any gear or pinion to the mass or inertia of the first pinion can be expressed as a function of the stage ratios and known constants. By further assuming: 1) the inertia of each gear and pinion is modeled as that of a solid cylinder with diameter equal to the pitch diameter of the gear, and 2) all gears and pinions are assumed to be constructed of the same material (i.e., all have the same material density), the “uniform maximum stress” pinion scaling condition can be expressed as follows:

$$\frac{J_{iP}}{J_{1P}}=\{\begin{array}{ccc} 1 & for & i=1\\ {\left(\prod_{k=1}^{i-1}s_{k}\right)}^{\frac{5}{3}} & for & i>1 \end{array}$$ (13)

$$\frac{J_{iG}}{J_{1P}}=\{\begin{array}{ccc} s_{i}{}^{4} & for & i=1\\ {\left(\prod_{k=1}^{i-1}s_{k}\right)}^{\frac{5}{3}}s_{i}{}^{4} & for & i>1 \end{array}$$ (14)

$$\frac{m_{iP}}{m_{1P}}=\{\begin{array}{ccc} 1 & for & i=1\\ \prod_{k=1}^{i-1}s_{k} & for & i>1 \end{array}$$ (15)

$$\frac{m_{iG}}{m_{1P}}=\{\begin{array}{ccc} s_{i}{}^{2} & for & i=1\\ \left(\prod_{k=1}^{i-1}s_{k}\right)s_{i}{}^{2} & for & i>1 \end{array}$$ (16)

Although more complex scaling assumptions could be adopted (e.g., by incorporating velocity considerations), the expressions given by (13–16) likely capture the most salient design relations employed in many multistage gear design problems. It should be noted, however, that different scaling assumptions (such as those for instrument gear trains or different types of transmissions) could be applied to the optimization formulation by adapting the right hand sides of (13–16).

## Examples and design implications

The optimization of (2), (5), and (7) subject to the constraint expressed in (1) was performed in MATLAB for a representative design scenario using the constrained nonlinear multivariate optimization tool, *fmincon*. The optimal stage ratios can be calculated with respect to each objective function (maximum efficiency, maximum acceleration, and minimum mass) for various numbers of stages and total transmission ratios (*n* and *N*). In the example presented here, a total transmission ratio of 250:1 was examined (*N* = 250). The gear geometry and friction parameters used in the analysis were taken from [26] and are as follows: *μ* = 0.05, *Z*_1_ = 12 (consistent with assumptions of minimum teeth on the pinion), *β* = 0, *ε*_*α*_ = 1.2, and Λ = 0.5. It should be noted that when the friction coefficient is set to zero, the gear efficiency is 100%, and the maximum acceleration optimization is the same as the minimum reflected inertia optimization performed in [2, 5, 6, 25]. For this example system, the stage ratio selection was evaluated for *n* = 3 to *n* = 10 to examine how different numbers of stages affected both the stage ratio selection and overall performance metrics (efficiency, acceleration, and mass). Transmissions with more than ten stages were not investigated since these designs exceed the optimal number of stages as determined by [2] for this design scenario.

### Implications regarding stage ratios

**Fig 2:**
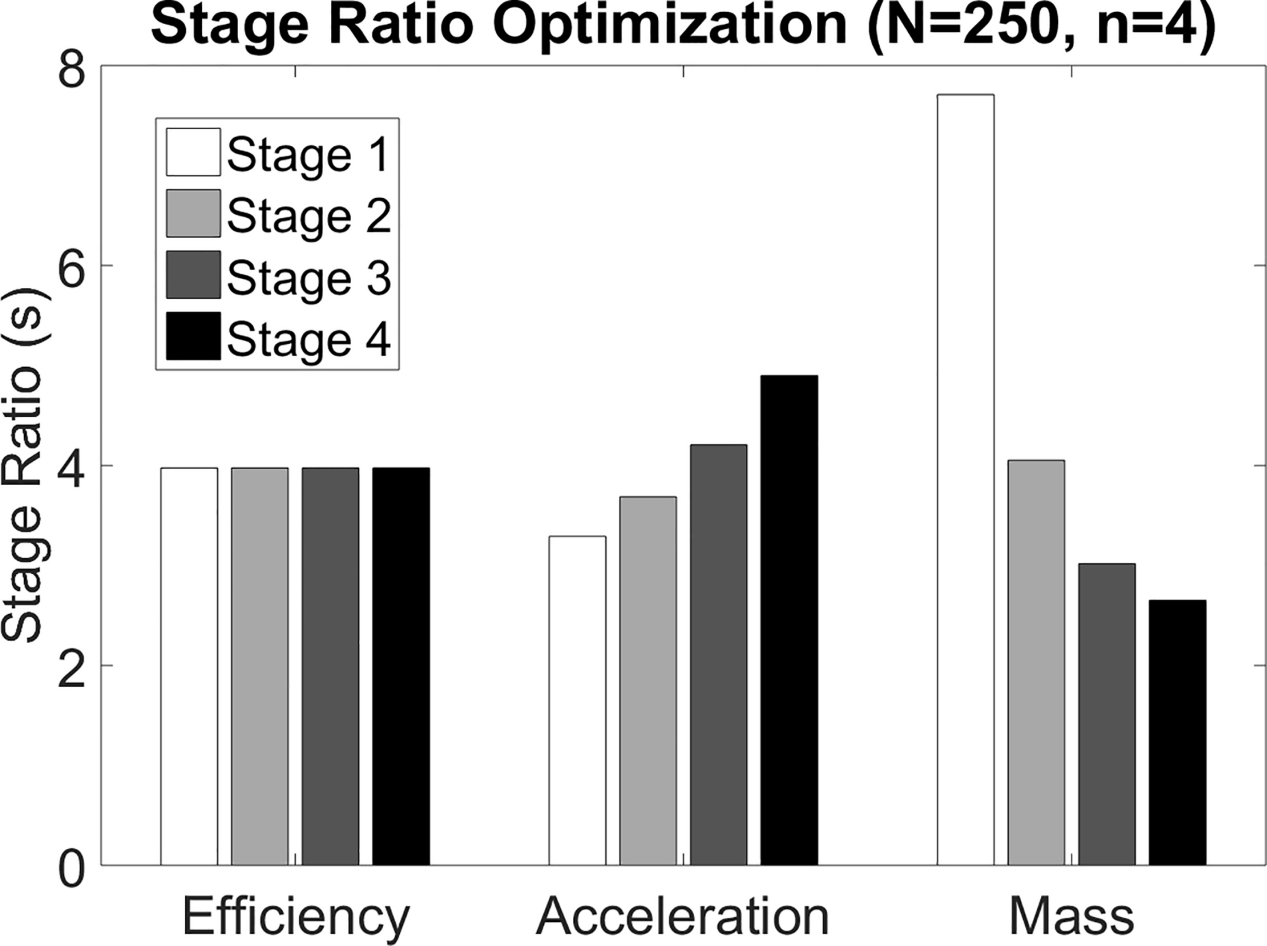
A bar plot showing the distribution of stage ratios for a 4-stage transmission with a total ratio of 250:1. The ratios of each of the 4 stages are selected to optimize one of three objective functions: efficiency, normalized acceleration, and normalized mass. The stage ratio values for these transmissions are shown as darker shades of gray moving from the motor to the load.

The optimal stage ratios with respect to the three objective functions for a 4-stage (*n* = 4) transmission with a total ratio of 250:1 can be seen in Fig 2. This figure depicts the trends in optimal stage ratios for the different optimization criteria. The authors note that these trends are consistent across large values of *N* common in multistage transmissions. Specifically, for purposes of achieving maximum efficiency, the stage ratios in each stage of the gear train should all be equal; for purposes of achieving maximum acceleration, the stage ratios of the various stages should increase monotonically from the motor towards the load; and for purposes of achieving minimal transmission mass, the stage ratios should decrease from the motor towards the load. There is, therefore, a direct tradeoff between mass minimization and acceleration maximization (i.e., essentially rotational inertia minimization) in the design of a multistage power gear transmission. This tradeoff is significant, as the minimum mass solution decreases the acceleration by 51% relative to its optimum, while the maximum acceleration solution increases the mass by 19% relative to its optimum, for the case (*n* = 4, *N* = 250) considered here.

### Implications regarding number of stages

**Fig 3:**
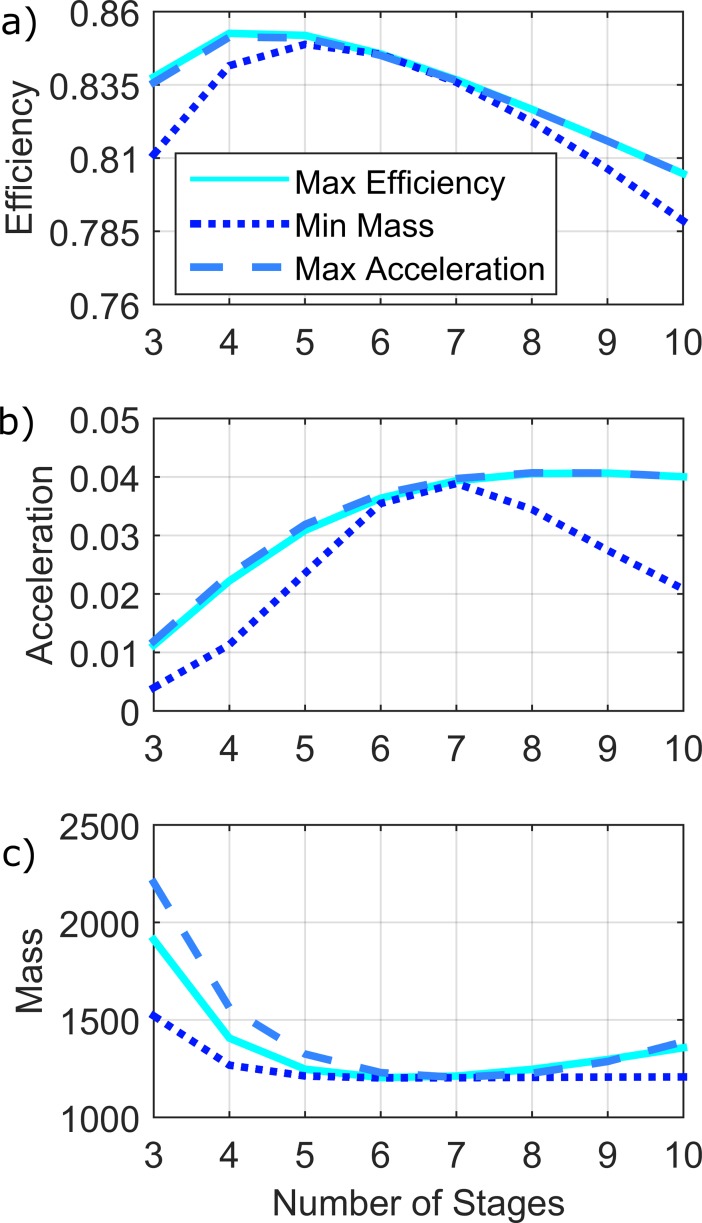
The objective function evaluations once the stage ratios have been selected to maximize efficiency (solid light blue), maximize normalized acceleration (dashed medium blue), or minimize normalized mass (dotted dark blue). The objective functions evaluated are transmission efficiency (a), normalized acceleration (b), and normalized mass (c).

For a desired total ratio of 250:1, stage ratios were optimized with respect to the three objective functions (efficiency, acceleration, and mass) for various values of *n* (3≤*n*≤10), and the efficiency, normalized acceleration capability, and normalized mass of the resulting transmissions were then calculated using (2), (5), and (7), respectively. The results of this analysis can be seen plotted against the number of stages in Fig 3. The transmissions optimized for efficiency, acceleration, and mass are plotted in solid light blue, dashed medium blue, and dotted dark blue respectively. This analysis lends insight into the selection of the number of stages in a transmission. Specifically, as can be seen in Fig 3A, the efficiency of the various transmissions peak at approximately 4–5 stages. The transmissions designed to maximize efficiency obtain higher efficiencies than the ones optimized for acceleration and mass, but the difference between the overall efficiency values is not substantial. In fact, the range of transmission efficiency obtainable by varying the number of stages in the transmission is also very small (approximately 6% change in efficiency from *n* = 3 to *n* = 10).

Fig 3B shows how the normalized acceleration properties vary with the number of stages in the transmission. As can be seen, adding additional stages to the transmission significantly increases the acceleration capabilities of the system up to a point, but an upper limit exists in all cases. In this example, the acceleration capabilities increase rapidly from 3 to 4 stages, but the performance increases become less significant as more stages are added. In fact, the local curvature of this objective function around the maxima (*n* = 8) is relatively flat, indicating that a designer could choose significantly fewer than the optimal number of stages without substantially compromising maximum acceleration.

Fig 3C shows that adding additional stages to a transmission can significantly decrease its mass, up to a point. It should be noted that the local curvature of Fig 3C is relatively flat around its minima (*n* = 6), but relatively steep between 3 and 5 stages indicating that the most of the improvements that can be made in terms of minimizing mass can be done with fewer than 6 stages. It should also be noted that when the stage ratios are selected to minimize mass, the transmission mass is significantly less than if the stage ratios are selected to maximize either of the other two objective functions, particularly for 3 or 4 stage transmissions.

It is important to note that when optimizing the stage ratios for efficiency, acceleration, or mass, the same general trends hold with respect to the number of stages in Fig 3A–3C. This indicates that the selection of the number of stages of a transmission has a larger effect on the performance metrics treated here than does the selection of the individual stage ratios of the transmission.

**Fig 4:**
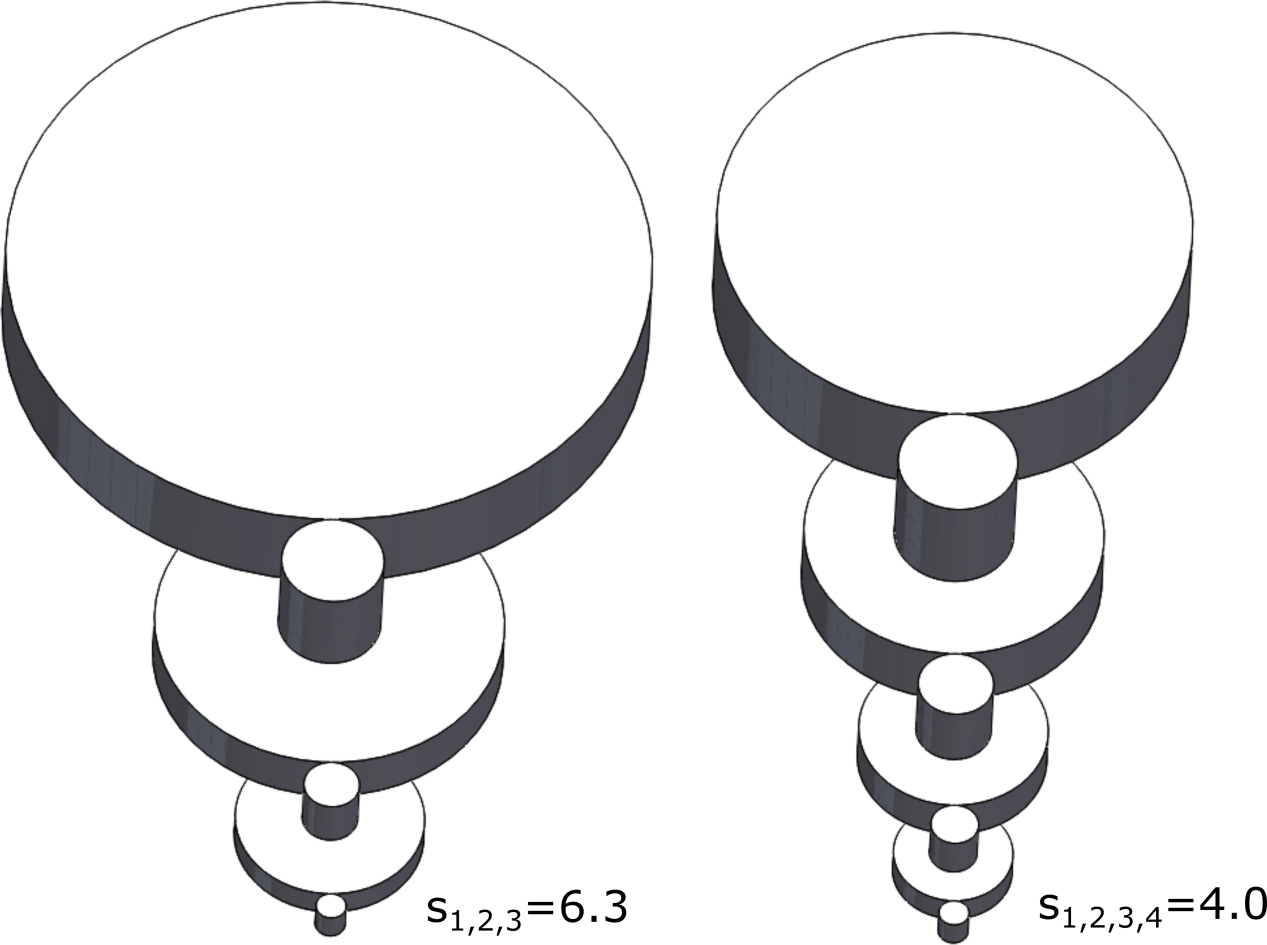
Physical depiction of a 3-stage (a) and a 4-stage (b) transmission with total ratios of 250:1 which maximize transmission efficiency. The 4-stage transmission has higher efficiency, higher normalized acceleration, and lower mass.

For the case of *N* = 250, a 3-stage and a 4-stage transmission, both optimized for maximum efficiency, are shown to scale in Fig 4A and 4B, respectively. The 3-stage transmission employs three equal stages of 6.3:1, while the 4-stage transmission employs four equal stages of 4.0:1 each. As indicated by the optimization, the 4-stage transmission reduces the overall mass by 26% relative to the 3-stage equivalent, and additionally increases the acceleration capabilities of the gear train by 50%. The 4-stage transmission also increases overall efficiency by 1%, which is obviously modest relative to the mass and acceleration advantages. Note that, since these gear trains are scaled by (12), the stress experienced by both gear trains is the same.

## Discussion

It is common for designers to use the minimum feasible number of stages when designing a multistage gear train. The analysis presented here, however, indicates that adding an additional stage (or stages) may significantly improve the performance of a drive system. An analytical solution for the number of stages to use in a multistage transmission to minimize the reflected inertia of the gear train was provided by [2]. However, that solution did not provide topological context for the optima. As can be seen from Fig 3, the local curvature around the optimum is small, which implies that a designer may select a number of stages for the transmission in the neighborhood of the optima without a large compromise in drive system performance. For this reason, the shape of these objective functions should be examined with respect to *n* so that design tradeoffs (such as mechanical complexity and packaging) may be effectively balanced.

As can be seen from Fig 3, similar trends hold with respect to the number of stages used in a transmission, regardless of how the stage ratios were selected. As such, all criteria are substantially affected by selection of number of stages, and therefore a designer should carefully consider the number of stages in the design process. Choosing the minimum feasible number of stages for the transmission may not provide optimal system performance, as indicated by Fig 3. As per the example described by Fig 4, although a 3-stage transmission may be feasible, the 4-stage equivalent provides improvements in transmission performance with respect to all three objective functions, regardless of which of the three selection criteria were used for selection of stage ratios.

With respect to selecting the individual stage ratios of a gear train, Fig 3 also indicates that stage ratio selection has a more significant impact on efficiency, acceleration, and mass when the number of stages in the transmission is low. This can be seen by the divergence of the three curves in Fig 3A–3C in the region of low number of stages. It is also interesting to note that the stage ratios show opposite trends when selected to minimize the mass of the transmission or to maximize the acceleration properties of a transmission, as shown in Fig 2. The divergence of optimal solutions highlights the value of considering the relative importance of minimal rotational inertia, versus minimum mass, when selecting the stage ratios for a given gear train and design application.

Considering multiple objective functions simultaneously, as is done in this work, allows a designer to assess which design objectives are sensitive to particular design parameters. For example, Fig 3 shows that the selection of number of stages has a significant effect on the acceleration capabilities of the drive system, but has a relatively small effect on the overall efficiency of the transmission. If maximizing efficiency were the only objective function considered, a designer may have overlooked the possibility of designing a transmission with maximum acceleration (with almost no cost in efficiency).

It should be noted that this optimization does not consider some aspects of transmission design that may affect the objective functions considered. For example, the mass of the shafts and bearings necessary to construct a transmission have not been considered, and the efficiency losses associated with bearings are not considered. The formulation presented here also does not include the effects of speed (i.e., pitch-line velocity) on gear stress. The volumetric packaging of the optimization’s resulting transmissions is also not considered, although this is expected to scale closely with transmission mass. Despite these limitations, the analyses presented here are believed to capture salient features of gear-based transmission design, and the formulation and implications of it should offer useful and accurate insights with respect to selecting the number of stages and stage ratios of gear trains for achieving desired performance characteristics.

## Conclusions

This paper describes a formulation that provides the optimal stage ratios for a *n*-stage multistage gear train with a desired overall transmission ratio that maximizes efficiency, maximizes transmission acceleration, or minimizes transmission mass, respectively. In addition to providing a formulation from which a designer can obtain the optimal number of stages and stage ratios, the authors highlight several related implications regarding gear train design, including the following two seemingly counterintuitive results. First, for a gear train that provides a given total gear reduction ratio, the respective objectives of minimizing rotational inertia (i.e., maximizing acceleration) and minimizing gear train mass are competing objectives with respect to selection of stage ratios. In particular, optimal stage ratio selection for a minimal rotational inertia solution entails successively increasing stage ratios, while optimal stage ratio selection a minimal mass solution entails successively decreasing stage ratios. Second, increasing the total number of stages can substantially decrease both mass and rotational inertia of a gear train. As such, substantially improved overall performance may be achieved by employing a number of stages that may be greater than the minimum feasible number of stages.

## Supporting information

S1 File MultistageOptimizationData. Matlab data file containing the output of the optimization run in this study.
(MAT)

S2 File Data Description. File explaining the data contained in MultistageOptimizationData.mat as well as the naming conventions used in this data set.
(PDF)
