# Supplementary material for: On the design of power gear trains: Insight regarding number of stages and their respective ratios
Source: PLoS One. 2018 Jun 1;13(6):e0198048. doi: 10.1371/journal.pone.0198048 (PMC5983518; doi:10.1371/journal.pone.0198048)
Supplement: S2 File — File explaining the data contained in MultistageOptimizationData.mat as well as the naming conventions used in this data set. (PDF) [file pone.0198048.s002.pdf]

Accompanying Data Description and Naming Convention

The accompanying data consists of the output of the optimization run in this study. Optimized stage ratios are included for N=250 and n=1 to n=10. Additionally, the mass, normalized acceleration, and efficiency of all optimized transmissions can be found in the data. The naming convention for all of the data is described below.

Data with s_X where X is either "acc", "eta", or "m" are cell arrays of stage ratio values. X indicates the objective function that generated those ratios (acc=acceleration, eta=efficiency, and m=mass). s_X(n) holds the optimal stage ratios for the objective function X where the number of stages is equal to n. Other data is stored in the form Y_X(n) where X is still the objective function as previously described and n is the number of stages of the transmission analyzed. For these data, Y indicates the evaluated metric of interest. For example, M_eta(5) indicates the normalized mass (Y=M) of a 5-stage (n=5) transmission optimized for maximum efficiency (X=eta).
